# Supplementary figures and images for: Cooperativity between the 3’ untranslated region microRNA binding sites is critical for the virulence of eastern equine encephalitis virus
Source: PLoS Pathog. 2019 Oct 28;15(10):e1007867. doi: 10.1371/journal.ppat.1007867 (PMC6936876; doi:10.1371/journal.ppat.1007867)

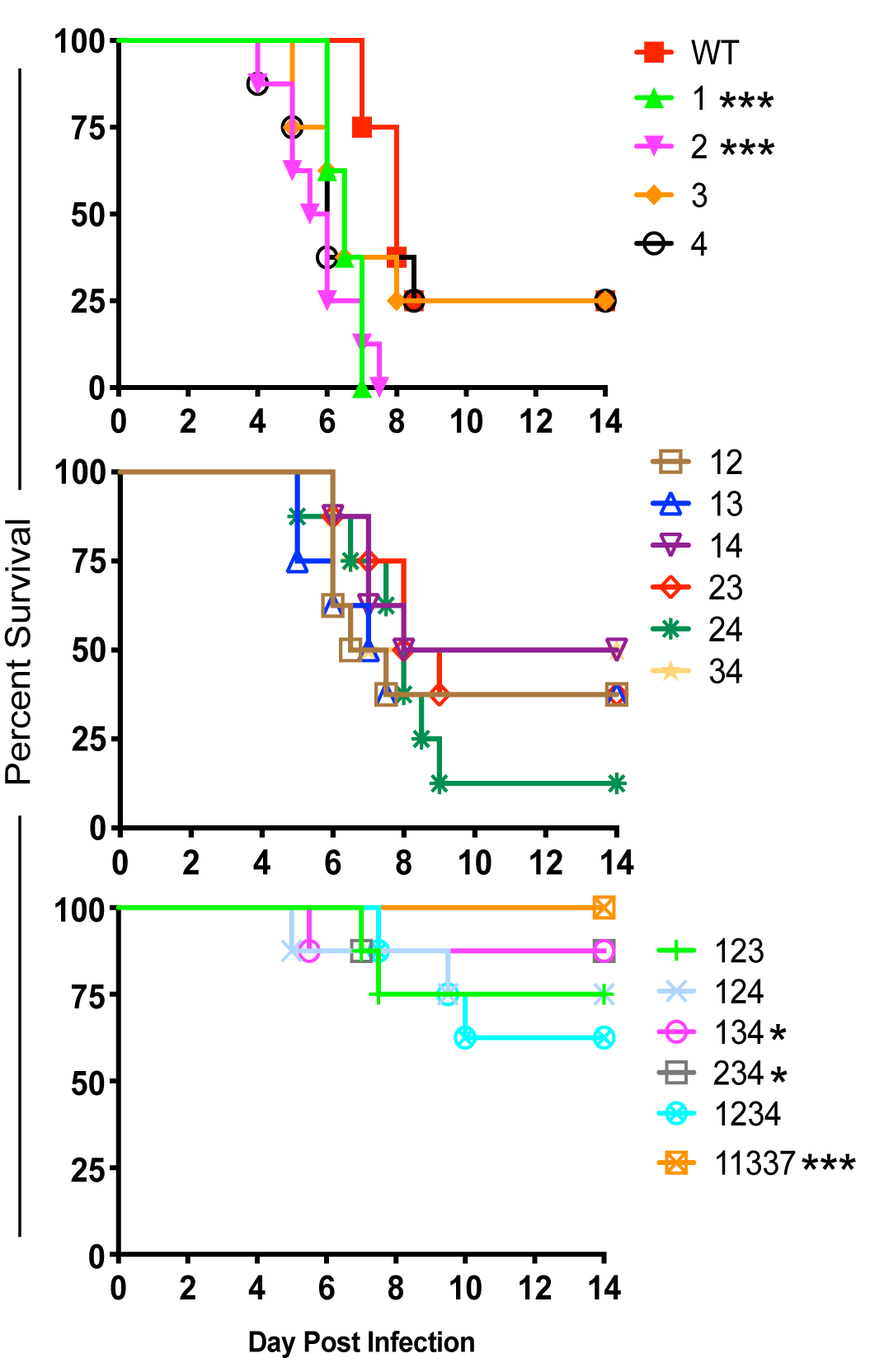

Supplement: S1 Fig — Female C57BL6 mice (5–6 weeks) were infected with 103 pfu sc in each footpad. Morbidity and mortality were measured twice daily. n = 7–8 mice from 2 independent experiments. (TIF) [file ppat.1007867.s001.tif]

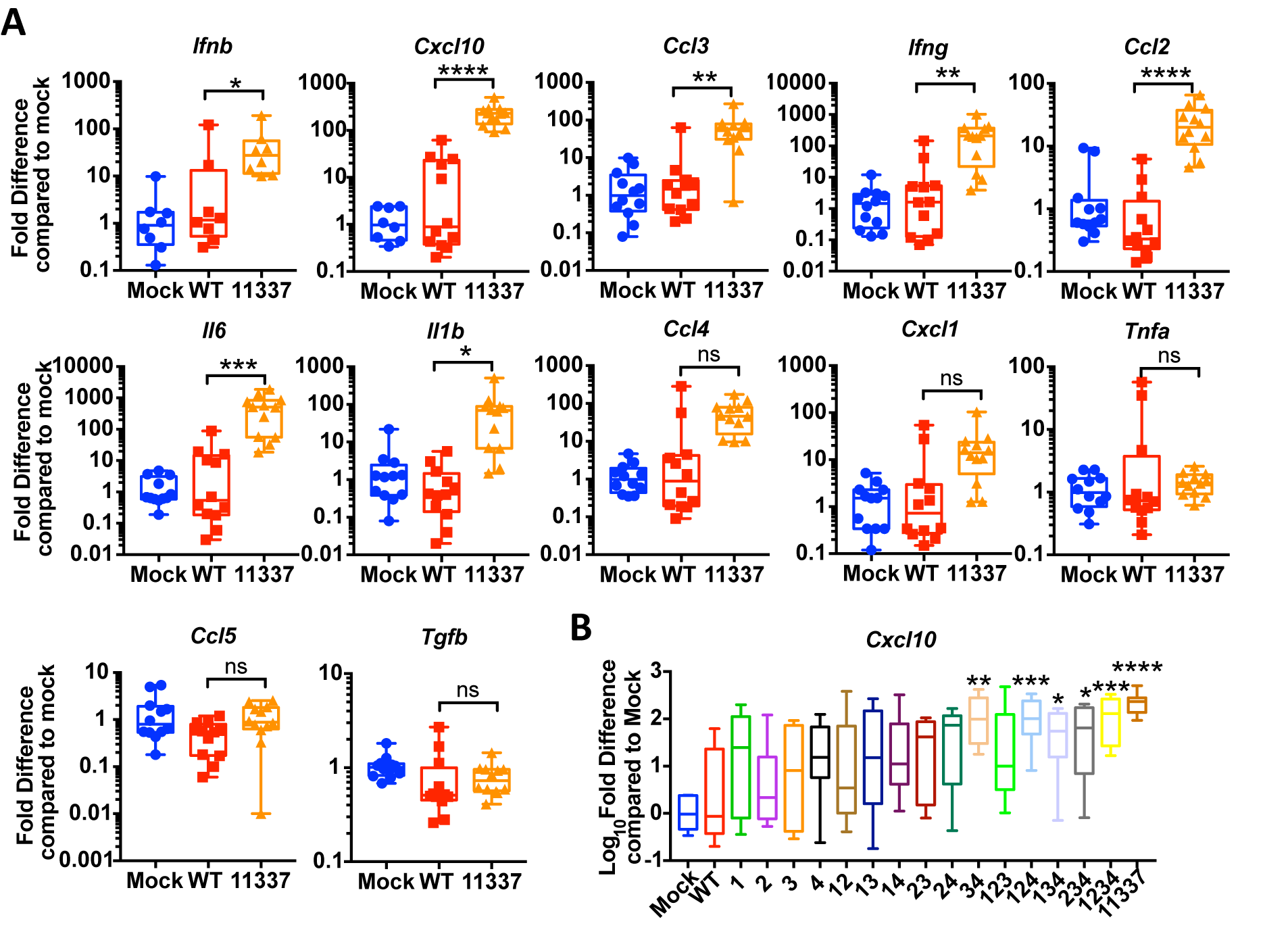

Supplement: S2 Fig — (A) Cytokine and chemokine mRNA levels in the PLN of CD-1 mice 12 hpi with 103 pfu of WT, 11337 or mock infected. Data is represented as fold difference compared to mock mice. n = 12 mice, 3 independent experiments. (B) Cxcl10 mRNA levels in PLN 12 hpi with EEEV mutants. n = 8–12 mice, 2–3 independent experiments *P<0.05, **P<0.01, ***P<0.001, ****P<0.0001, (A) one way analysis of variance test with corrections for multiple comparisons using Turkey method or (B) one way analysis of variance test between WT and each mutant with corrections for multiple comparisons using Holm-Sidak method of the log-transformed data. ns = non-significant. Box-and whisker plots represent min-max with bar representing the median value. (TIF) [file ppat.1007867.s002.tif]

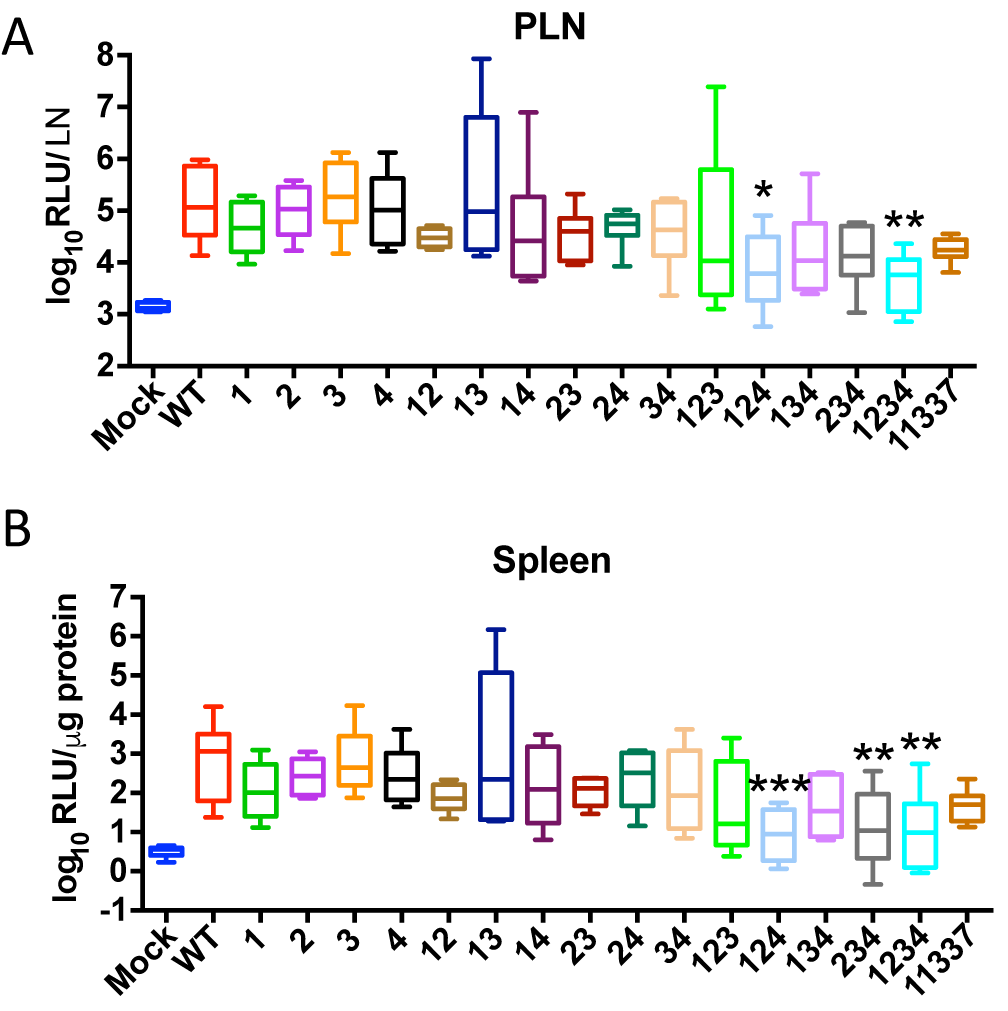

Supplement: S3 Fig — CD-1 mice were infected with 103 pfu of the EEEV mutants sc in each footpad. Tissues were harvested at 96 hours post infection. Virus replication in PLN (A), spleen (B). N = 8 mice, from 2 independent experiments. *P<0.5, **P<0.01, ***P<0.001, ****P<0.0001 one way analysis of variance test with corrections for multiple comparisons using the Holm-Sidak method comparing each mutant to WT. Box-and whisker plots represent min-max with bar representing the median value. (TIF) [file ppat.1007867.s003.tif]

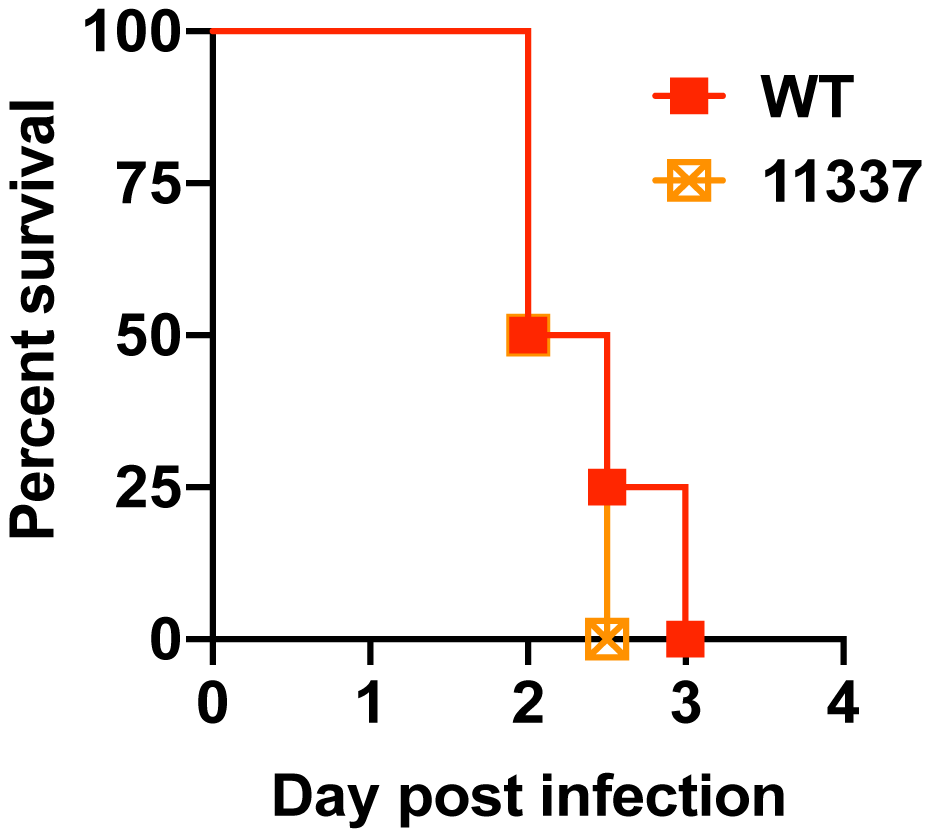

Supplement: S4 Fig — Survival of female (5–6 week) CD-1 infected with ic with either 103 pfu of WT or 11337 mutant. Morbidity and mortality were measured twice daily. n = 8 mice from 2 independent experiments. (TIF) [file ppat.1007867.s004.tif]

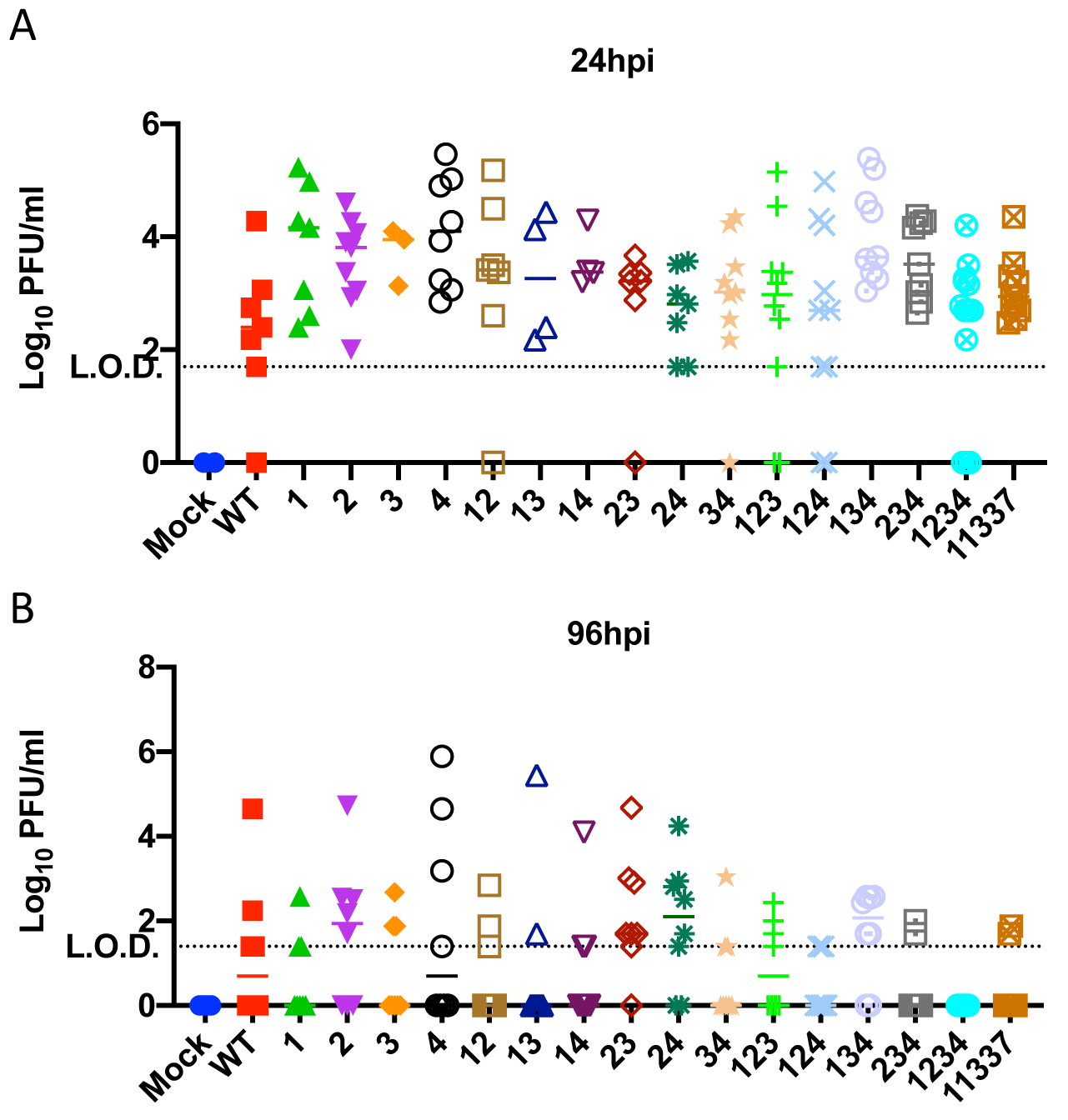

Supplement: S5 Fig — CD-1 mice were infected with 103 pfu of the EEEV mutants sc in each footpad. Serum was harvested at 24 hpi (A) and 96 hpi (B). n = 3–13 mice, from 2 independent experiments. L.O.D = limit of detection. No significant difference in serum titers was detected using one-way analysis of variance test with corrections for multiple comparisons using the Holm-Sidak method comparing each mutant to WT. Each point represents a single mouse. (TIF) [file ppat.1007867.s005.tif]

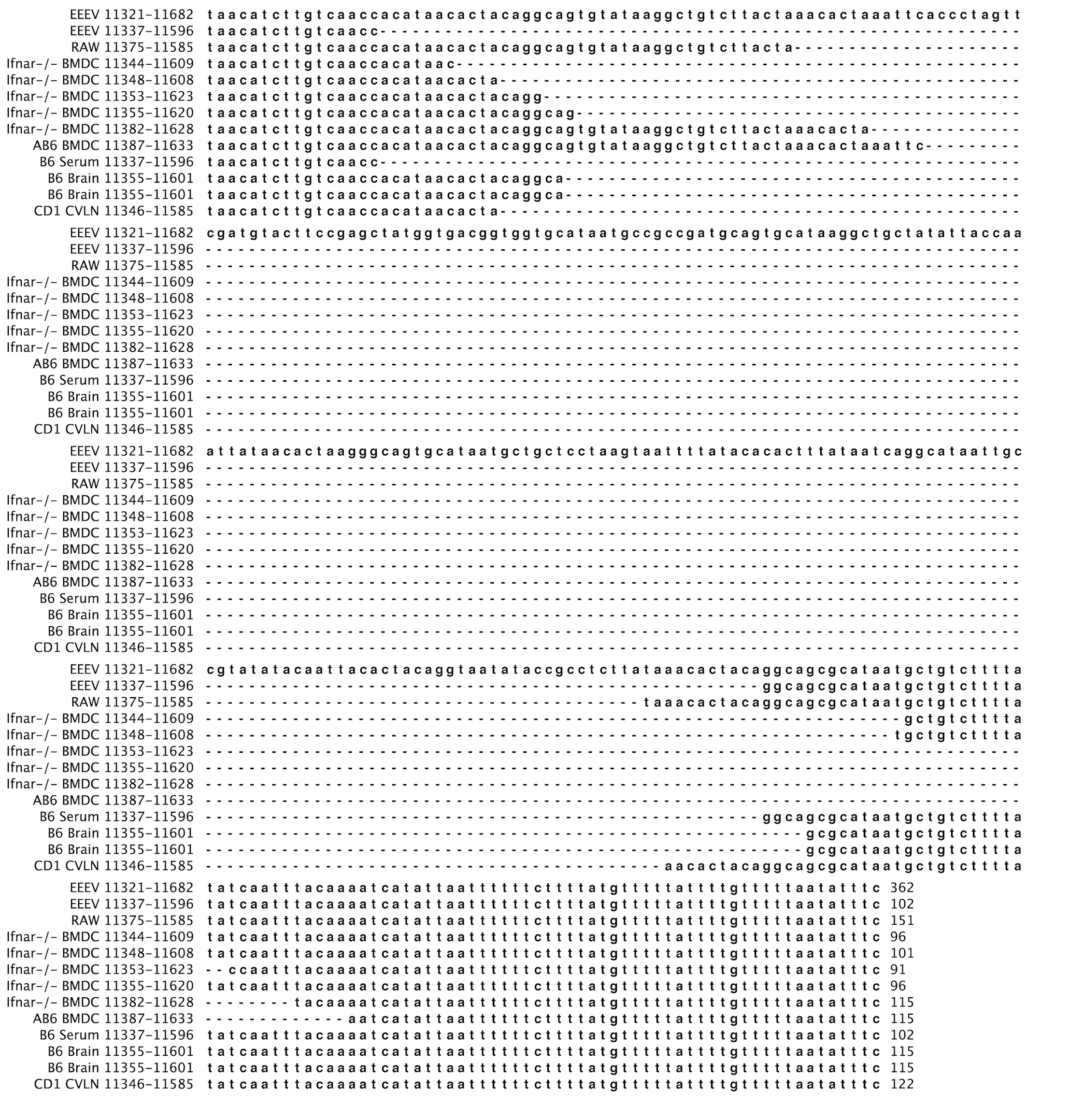

Supplement: S6 Fig — Alignment of escape mutants isolated for indicated cells or tissues. Numbers on left indicate location in the genome of the deletion. Numbers at end of the sequence indicate the length of the 3’ UTR in each escape mutant. B6—C57BL6, BMDC- bone marrow derived dendritic cell, CVLN–cervical lymph nodes. (TIF) [file ppat.1007867.s006.tif]
