# Supplementary material for: Cooperativity between the 3’ untranslated region microRNA binding sites is critical for the virulence of eastern equine encephalitis virus
Source: PLoS Pathog. 2019 Oct 28;15(10):e1007867. doi: 10.1371/journal.ppat.1007867 (PMC6936876; doi:10.1371/journal.ppat.1007867)
Supplement: S1 Table — (PDF) [file ppat.1007867.s007.pdf]

Primer	Sequence
EEEV ΔmiR-142-1-F	5′-taacatcttgtcaaccacataactcaagaggcagtgta-3′
EEEV ΔmiR-142-1-R	5′-attgtagaacagttggtgtattgagttctccgtcacat-3′
EEEV ΔmiR-142-2-F	5′-taaggctgtcttactaaactcaagattcaccctag-3′
EEEV ΔmiR-142-2-R	5′-ctagggtgaatcttgagtttagtaagacagcctta-3′
EEEV ΔmiR-142-3-F	5′-gcataattgccgtatatacaattactcaagaggtaatataccgcctcttataaa-3′
EEEV ΔmiR-142-3-R	5′-tttataagaggcggtatattacctcttgagtaattgtatatacggcaattatgc-3′
EEEV ΔmiR-142-4-F	5′-ataccgcctcttataaactcaagaggcagcgc-3'
EEEV ΔmiR-142-4-R	5′-gcgctgcctcttgagtttataagaggcggtat-3′
WEEV McM 11224-S	5′-cttataaacacacgtagatgactgagcgcggccatacacactttataaattcttttat-3′
WEEV McM 11224-AS	5′-ataaaagaatttataaagtgtgtatggccgcgctcagtcatctacgtgtgtttataag-3′
