# Supplementary material for: Cooperativity between the 3’ untranslated region microRNA binding sites is critical for the virulence of eastern equine encephalitis virus
Source: PLoS Pathog. 2019 Oct 28;15(10):e1007867. doi: 10.1371/journal.ppat.1007867 (PMC6936876; doi:10.1371/journal.ppat.1007867)
Supplement: S2 Table — (PDF) [file ppat.1007867.s008.pdf]

Primer	Forward Primer	Reverse Primer
18s	CGCCGCTAGAGGTGAAATTCT	CGAACCTCCGACTTTCGTTCT
Ifnb	GAACATTCGGAAATGTCAGG	ACTGTCTGCTGGTGGAGTTC
Cxcl10	GCCGTCATTTTCTGCCTCA	CGTCCTTGCGAGAGGGATC
Ccl3	TGAAACCAGCAGCCTTTGCTC	AGGCATTCAGTTCCAGGTCAGTG
Ifng	CAAAAGGATGGTGACATGAA	TTGGCAATACTCATGAATGC
Ccl2	CTTCTGGGCCTGCTGTTCA	CCAGCCTACTCATTGGGATCA
Il6	TTCCATCCAGTTGCCTTCTT	CAGAATTGCCATTGCACAAC
Il1b	CAGGCAGGCAGTATCACTCA	AGGCCACAGGTATTTTGTCG
Cxcl1	CAGCCACCCGCTCGCTTCTC	CAAGGCAAGCCTCGCGACCAT
Ccl5	AGATCTCTGCAGCTGCCCTCA	GGAGCACTTGCTGCTGGTGTAG
Tnfa	CCCACTCTGACCCCTTTACT	TTTGAGTCCTTGATGGTGGT
Ccl4	CCATGAAGCTCTGCGTGTCTG	GGCTTGGAGCAAAGACTGCTG
Tgfb	TGACGTCACTGGAGTTGTACGG	GGTTCATGTCATGGATGGTGC
